# Associations between biomarkers of environmental enteric dysfunction and oral rotavirus vaccine immunogenicity in rural Zimbabwean infants

**DOI:** 10.1016/j.eclinm.2021.101173

**Published:** 2021-11-15

**Authors:** James A Church, Sandra Rukobo, Margaret Govha, Ethan K Gough, Bernard Chasekwa, Benjamin Lee, Marya P Carmolli, Gordana Panic, Natasa Giallourou, Robert Ntozini, Kuda Mutasa, Monica M McNeal, Florence D. Majo, Naume V. Tavengwa, Jonathan R. Swann, Lawrence H Moulton, Beth D Kirkpatrick, Jean H Humphrey, Andrew J Prendergast

**Affiliations:** a Zvitambo Institute for Maternal and Child Health Research, Harare, Zimbabwe; b Centre for Genomics and Child Health, Blizard Institute, Queen Mary University of London, 4 Newark Street, London E1 2AT, UK; c Departments of Pediatrics, Vaccine Testing Center, Larner College of Medicine, University of Vermont, Burlington, VT, USA; d Departments of Microbiology and Molecular Genetics, Vaccine Testing Center, Larner College of Medicine, University of Vermont, Burlington, VT, USA; e Faculty of Medicine, Imperial College London, South Kensington Campus, London SW7 2AZ, UK; f Department of Pediatrics, University of Cincinnati College of Medicine, Division of Infectious Diseases, Cincinnati Children's Hospital Medical Center, Cincinnati, OH, USA; g Department of International Health, Johns Hopkins Bloomberg School of Public Health, Baltimore, MD, USA

**Keywords:** Infants, Africa, Rotavirus, Oral vaccine, Enteropathy, Environmental enteric dysfunction

## Abstract

**Background:**

Oral rotavirus vaccines (RVV) are poorly immunogenic in low-income countries. Environmental enteric dysfunction (EED) resulting from poor water, sanitation and hygiene (WASH) may contribute. We therefore tested associations between EED and RVV immunogenicity, and evaluated the effect of improved WASH on EED.

**Methods:**

We measured nine biomarkers of EED among Zimbabwean infants born to mothers enrolled in a cluster-randomised 2 × 2 factorial trial of improved WASH and improved feeding between November 2012 and March 2015 (NCT01824940). We used multivariable regression to determine associations between EED biomarkers and RVV seroconversion, seropositivity and geometric mean titer. Log-binomial regression was used to evaluate the effect of improved WASH on EED.

**Findings:**

Among 303 infants with EED biomarkers and immunogenicity data, plasma intestinal fatty-acid binding protein and stool myeloperoxidase were positively associated with RVV seroconversion; adjusted RR 1.63 (95%CI 1.04, 2.57) and 1.29 (95%CI 1.01, 1.65), respectively. There were no other associations between RVV immunogenicity and either individual biomarkers or EED domains (intestinal permeability, intestinal damage, intestinal inflammation and microbial translocation). EED biomarkers did not differ between randomised WASH and non-WASH groups.

**Interpretation:**

We found no evidence that EED was associated with poor RVV immunogenicity. Contrary to our hypothesis, there was weak evidence that EED was associated with increased seroconversion. EED biomarkers were not affected by a package of household-level WASH interventions.

**Research in context**

**Evidence before this study**

Environmental enteric dysfunction (EED) is a near ubiquitous disorder of small intestinal structure and function in low- and middle-income countries (LMIC), which has been hypothesised to contribute to reduced oral vaccine performance. A recent systematic review evaluating EED and oral vaccine responses identified eight relevant studies and concluded that existing evidence is insufficient to determine whether EED contributes to oral vaccine underperformance. We performed an updated literature search using *Medline* to identify articles published up to 31st December 2020, using the search terms vaccine or immunisation and enteropathy or enteric dysfunction, but found no new articles describing vaccine responses in the context of EED.

**Added value of this study**

This study capitalised on a large and well-characterised birth cohort of rural Zimbabwean infants and evaluated an extensive panel of gut biomarkers around the time of oral vaccine administration. It is the first study to explore relationships between several novel biomarkers and rotavirus vaccine immunogenicity, providing new insights into the relationship between EED and oral vaccine performance.

**Implications of all the available evidence**

Our findings showed no deleterious impact of EED biomarkers on oral rotavirus vaccine responses. Interventions targeting EED may therefore not be effective in improving oral RVV performance, and further studies are needed to better understand the impact of the intestinal milieu on oral vaccine immunogenicity.

Alt-text: Unlabelled box

## 1 Introduction

Increased coverage of oral rotavirus vaccines (RVV) has contributed to global declines in diarrheal disease burden [1]. However, these vaccines fail to reach their full potential in regions of high child mortality. In sub-Saharan Africa, one-year efficacy of monovalent RVV against severe rotavirus gastroenteritis was 61.2% [2] compared to 84.7% efficacy for the same vaccine in Latin America and Finland [3]. Data on vaccine immunogenicity mirror this efficacy gap with mean seroconversion of 53% versus 87% in high and low-mortality countries, respectively [4].

The reasons RVV performs so differently across settings are unclear, however intestinal factors may contribute [5], including a subclinical disorder termed environmental enteric dysfunction (EED). EED comprises several aberrations in small intestinal physiology and integrity, characterised by villous blunting, crypt hyperplasia and lymphocytic infiltration [6]. These alterations in gut structure and function develop early in life in impoverished settings [7,8] and may interfere with the processing of RVV as it transits through the small intestine.

Given the challenges of obtaining small intestinal biopsies in infants, most studies of EED rely on non-invasive intestinal biomarkers. Approaches range from using a single biomarker as the primary outcome for EED (such as percentage urinary lactulose excretion following oral ingestion [9]) to EED scores, which combine multiple biomarkers [10,11]. Among studies exploring associations between EED and oral vaccine immunogenicity, results have been heterogeneous [12]. In addition, a lack of consensus on the etiology, definition and measurement of EED further complicates our understanding of how EED might reduce oral vaccine performance.

We recently reported findings from a cluster-randomised trial of improved water, sanitation and hygiene (WASH) in rural Zimbabwe. Infants randomised to WASH, compared to non-WASH, had 50% greater RVV seroconversion (from approximately 20% to 30%) [13]. We hypothesised that the WASH intervention reduced EED, thereby increasing RVV immunogenicity. To explore this, we measured a range of stool and plasma biomarkers to determine (1) the association between EED biomarkers and RVV immunogenicity (defined as RVV seroconversion, seropositivity and IgA titre); and (2) the effect of improved WASH on EED biomarkers around the time of rotavirus vaccination.

## 2 Methods

The infants selected for the current study comprised a subgroup of infants recruited into the Sanitation Hygiene Infant Nutrition Efficacy (SHINE) trial, which was a 2 × 2 factorial cluster-randomised trial in two rural districts in Zimbabwe assessing the independent and combined effects of improved WASH and improved infant and young child feeding (IYCF) on stunting and anemia (NCT01824940; registration 05/04/2013) [14]. The full trial protocol is available online at https://osf.io/w93hy. Women becoming pregnant between November 2012 and March 2015 were eligible if they lived in clusters randomised to: Standard-of-care (SOC), IYCF, WASH, or combined IYCF plus WASH. The trial found a modest effect of IYCF on linear growth and hemoglobin at 18 months of age, but no effect of WASH on either outcome [15]. Neither intervention reduced diarrhea.

### 2.1 Rotavirus vaccination

In May 2014, oral monovalent rotavirus vaccine (*Rotarix*™) was introduced in Zimbabwe and given with oral polio vaccine at 6 and 10 weeks of age. Vaccination was undertaken at local clinics and not overseen by the trial; however, national rotavirus vaccination coverage in 2015–2016 was 87–91% [16]. Trial staff recorded vaccination dates by reviewing child health cards. Each child's *Rotarix* vaccination status was categorised as complete (two doses), incomplete (one dose) or unvaccinated.

### 2.2 Substudy population

From June 2014, infants enrolled into this substudy had blood and stool collected at 1, 3, 6, 12 and 18 months of age [17]. Substudy infants were eligible for the current analysis if they were HIV-unexposed, had a post-rotavirus vaccination IgA titre, and an available plasma or stool sample collected before six months of age. Infants with missing rotavirus vaccination data, or without documented receipt of at least one dose of RVV, were excluded. For the RVV EED analysis, we also excluded EED measurements post-vaccination, since vaccine administration itself may induce intestinal changes [18].

### 2.3 Anti-rotavirus IgA assay

Plasma anti-rotavirus IgA, the most widely-used marker of oral rotavirus vaccination [19], was measured by Enzyme Linked Immunosorbent Assay (ELISA), as previously described [20]. Seroconversion was defined as a post-vaccine plasma concentration of anti-rotavirus IgA ≥20 U/mL in infants who were seronegative (<20 U/mL) pre-vaccination [21]. Seropositivity was defined as a post-vaccine titer ≥20 U/mL, regardless of pre-vaccine titer.

### 2.4 EED biomarkers

A range of biomarkers were selected to characterise four domains of EED [12]. Briefly, we measured markers of (1) intestinal inflammation (stool neopterin and myeloperoxidase), (2) small intestinal damage and repair (plasma intestinal fatty acid binding protein (I-FABP), plasma citrulline, stool regenerating gene 1β (REG-1B)), (3) intestinal permeability (stool alpha-1 antitrypsin (A1AT)), and (4) microbial translocation and systemic inflammation (plasma soluble CD14, kynurenine:tryptophan ratio (KTR), and C-reactive protein (CRP)). Plasma samples were tested by ELISA according to manufacturers’ instructions for CRP (limit of detection (LOD) 0.01 ng/mL), soluble CD14 (LOD 125pg/mL) (both R&D Systems, Minneapolis, MN, USA); and I-FABP (LOD 47pg/mL) (Hycult Biotechnology, Uden, The Netherlands). Plasma citrulline (LOD 100 ng/mL), kynurenine (LOD 40 ng/mL), and tryptophan (200 ng/mL) were assayed by ultrahigh-performance liquid chromatography tandem mass spectrometry with electrospray ionisation (Waters, Wilmslow, U.K.) at Imperial College, London.

Stool samples were tested by ELISA according to manufacturers’ instructions for neopterin (LOD 0.7 nmol/L; GenWay Biotech Inc, San Diego, USA), myeloperoxidase (LOD 1.6 ng/mL; Immundianostik, Bensheim, Germany), A1AT (LOD 1.5 ng/mL; BioVendor, Brno, Czech Republic), and REG‐1β (LOD 0.625 ng/mL; TECHLAB Inc, Blacksburg, USA).

### 2.5 EED definition

Given the absence of an established case definition, we used several different definitions of EED. Firstly, biomarkers were examined individually. Secondly, biomarkers were grouped according to the four domains detailed above [12]. For each domain, composite scores were created using tertile categories from biomarkers in that domain, with categories defined as 0 (<25th centile), 1 (25th–75th percentile), or 2 (>75th percentile). Table S1 details the range of scores possible for each domain. For each infant, valid data were required for all biomarkers to generate a domain score. Thirdly, we carried out a principal components analysis to identify the most informative combinations of biomarkers. Among infants with data on all 9 biomarkers, a scree plot was generated to select the appropriate number of principal components. Components with an *Eigen* value greater than one were considered meaningful [22]. To guide interpretation and identify relevant biological domains, the loading of biomarkers was evaluated, using a cut-off of ≥0.4 in absolute value to identify the biomarkers contributing most to each principal component. Finally, we generated two composite EED scores. The first replicated the score developed by Kosek and colleagues using three stool biomarkers (AAT, MPO and NEO) [10]. The second score (SHINE EED score) was calculated from the sum of tertile scores (described above) for all nine available biomarkers, with the sum ranging from 0 to 18.

### 2.6 Statistical analyses

For all analyses, the primary outcome was rotavirus vaccine seroconversion among infants at the individual level; secondary outcomes were rotavirus vaccine seropositivity and the anti-rotavirus IgA geometric mean titre (GMT). All biomarkers were natural-log converted to reduce skewness. To handle outliers, resulting from intercurrent illness for example, biomarker variables were transformed through 90% winsorisation (i.e. truncation at the 5th and 95th centiles) [23].

First, we tested individual biomarkers as continuous explanatory variables. Multivariable generalised estimating equations (GEE) were used with a log-binomial link to estimate a risk ratio (RR) of seroconversion/seropositivity, or an identity link to estimate the ratio of GMT per log increase in each biomarker. In adjusted analyses, we included intervention arm (WASH versus non-WASH), season of birth, breastfeeding status and weight-for-age *Z*-score around the time of vaccination, based on biological plausibility. To handle zero-inflated semi-continuous GMT data, we used a log-normal censored regression model (Tobit), with left censoring at 7.5 U/mL (the lower limit of anti-RV IgA detection). Multiple comparisons were accounted for using the Benjamini-Hochberg procedure [24]. Secondly, we tested associations between grouped measures of EED and RVV immunogenicity. Domains were expressed either in dichotomous form (presence/absence of one or more biomarkers in the top quartile for a given domain) or categorical (cumulative score of quartiles in a given domain). Principal components were expressed as continuous scores. Thirdly, we performed a sensitivity analysis including infants with EED measured post-RVV receipt.

Finally, we estimated the effects of improved WASH on EED around the time of RVV receipt by including all infants with available biomarker data measured before and after RVV receipt. Infants with biomarkers measured after RVV receipt were included (a) to increase sample size and (b) because the host immune response to RVV is dynamic and intestinal changes in the period after vaccine receipt may still be important. These analyses were intention-to-treat at the child level. We collapsed trial arms into WASH (WASH and WASH+IYCF) versus non-WASH (SOC and IYCF) since the IYCF intervention started at 6 months of age (beyond the window for rotavirus vaccination and stool collection). Except for citrulline, the effect of WASH was evaluated by fitting a separate tobit regression model, to account for biomarker values below the LOD, with each biomarker as the dependent variable. Sandwich standard error estimation was used to account for cluster membership. For citrulline, no infants were below the LOD, so linear regression models were fitted by GEE, with an exchangeable correlation to account for cluster membership. Regression coefficients were reported as the ratio of biomarker concentrations between intervention and control arms.

Tobit regression models for the effect of WASH on EED biomarkers were fitted using the package *AER* [25] and sandwich standard errors were estimated with the *sandwich* package [26]; while the *geepack* package was used to fit models for the effect of WASH on Citrulline by GEE [27], all in R version 3.5.3. All other statistical analyses were performed using STATA version 14 (College Station, TX: StataCorp LP) and Prism v7 (GraphPad Software Inc., CA, USA).

### 2.7 Ethical approval

The original SHINE trial and the rotavirus immunogenicity sub-study were approved by the Medical Research Council of Zimbabwe and Johns Hopkins Bloomberg School of Public Health Committee on Human Research. Written informed consent was obtained from all mothers prior to enrolment in the main trial and the EED substudy. All experimental protocols were conducted in accordance with the World Medical Association Declaration of Helsinki and reporting of this sub-study adheres strictly to STROBE guidelines.

### 2.8 Role of funding sources

This work was supported by the Wellcome Trust [203,905/Z/16/Z to JAC and 093,768/Z/10/Z and 108,065/Z/15/Z to AJP]. The SHINE trial was funded by the Bill & Melinda Gates Foundation [OPP1021542 and OPP113707]; UK Department for International Development (UK Aid); Swiss Agency for Development and Cooperation and US National Institutes of Health [2R01HD060338-06]. The study funders approved the trial design, but were not involved in data collection, analysis, interpretation, or manuscript preparation.

## 3 Results

### 3.1 Baseline characteristics

**Fig. 1:**
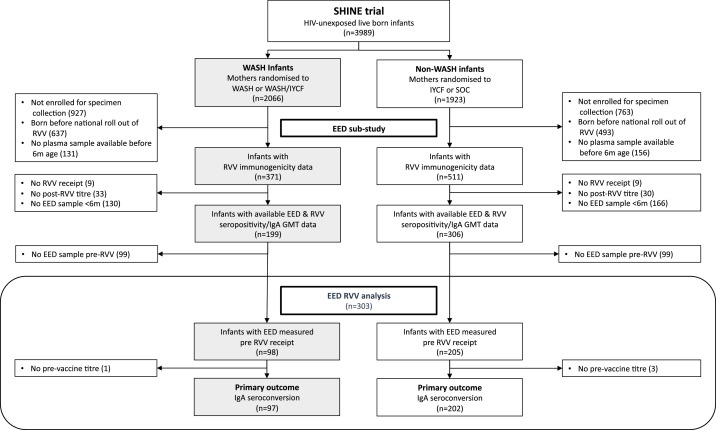
CONSORT diagram showing selection of infants for rotavirus sub-study and subsequent EED biomarker analysis. WASH = Water, sanitation & hygiene; EED = environmental enteric dysfunction, IYCF = infant & young child feeding; SOC = standard of care; RVV = rotavirus vaccine; GMT = geometric mean titre.

Among 882 infants with anti-rotavirus IgA titres (371 WASH and 511 non-WASH infants), 505 (57%) had both RVV immunogenicity data and a valid EED biomarker measured in the first six months after birth. Of these, 303 (34%) had biomarkers measured before the first dose of Rotarix and were included in the main analysis (98 WASH and 205 non-WASH infants) (Fig. 1). Table S2 outlines the characteristics of these infants, together with baseline maternal and household variables, compared to the overall trial population. Among the subgroup of 303 infants with EED measured pre-RVV, 37% were born in the rotavirus season. Low birthweight (<2.5 kg) affected 6% of infants; 95% of infants were being exclusively breastfed at three months of age. Plasma biomarkers were available for 300 (99%) infants and stool biomarkers for 264 (87%) infants. The median timing of pre-vaccine titre measurement was 10 days (IQR 6, 14) prior to the first dose of rotavirus vaccine, and for post-vaccine titre measurement was 27 days (IQR 18, 41) after the last dose of rotavirus vaccine. Among all 303 infants in the main analysis, seroconversion and seropositivity rates were 24.4% and 26.1%, respectively, and there were no meaningful differences in infant characteristics between RVV seroconverters and non-seroconverters (Table S3a). The median infant age at EED specimen collection was 35 days (IQR 32, 38), corresponding to a median of 10 days (IQR 6, 14) before the first RVV dose.

### 3.2 Overall burden of EED

Mean individual biomarker concentrations were high compared with normal ranges derived from healthy cohorts in high-income countries [28], [29], [30], [31]. When biomarkers were divided into quartiles, 221 (73%) infants had at least one biomarker in the top quartile. Among 146 infants with measurements across all nine biomarkers, 53 (36%) infants had 3 or more biomarkers in the top quartile, and 47 (32%) infants scored in the top quartile for two or more biomarker domains (Fig. S1). Baseline characteristics among the 146 infants with full biomarker data were comparable to the 157 infants with incomplete biomarker data (Table S3b)

### 3.3 Associations between EED and RRV immunogenicity

**Fig. 2:**
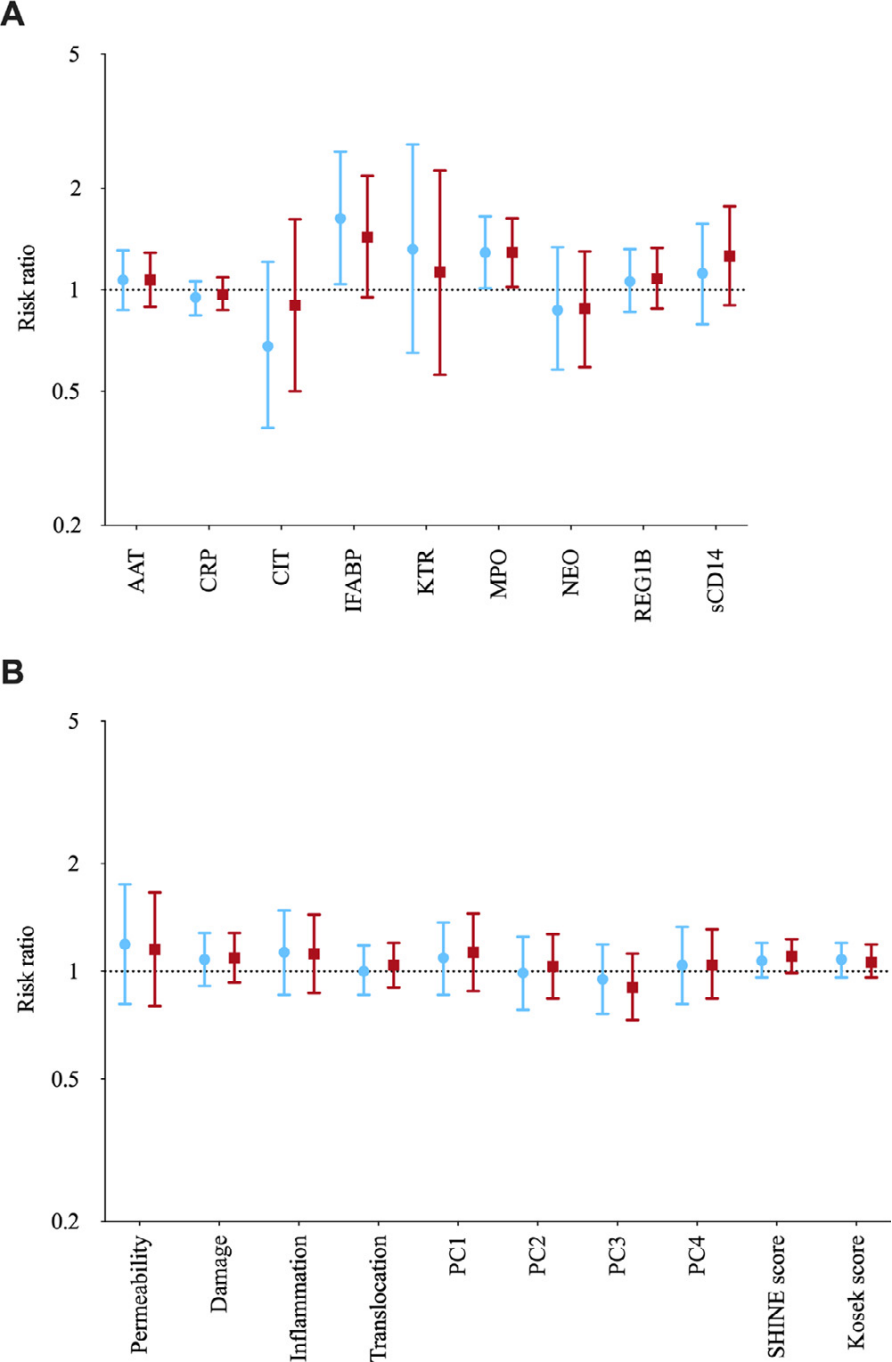
Associations between (A) individual EED biomarkers and (B) EED biomarkers domains and scores, and oral rotavirus vaccine immunogenicity. Risk ratios for seroconversion/seropositivity correspond to a 1 natural log increase. Risk ratios and 95% confidence intervals shown are for the adjusted analysis. Blue = seroconversion, Red = seropositivity. AAT = alpha-1 antitrypsin, CRP = C-reactive protein, CIT = citrulline, IFABP = intestinal fatty acid binding protein, KTR = kynurenine tryptophan ratio, MPO = myeloperoxidase, NEO = neopterin, REG1B = regenerating enzyme 1B, sCD14 = soluble CD14, PC = principal component. (For interpretation of the references to color in this figure legend, the reader is referred to the web version of this article).

Overall, there were few meaningful associations between individual EED biomarkers and RVV immunogenicity. In unadjusted analyses, I-FABP was positively associated with rotavirus seroconversion (RR 1.70 (95%CI 1.06, 2.73) per log-rise I-FABP) (Table 1). In adjusted analyses, elevated I-FABP remained positively associated with RVV seroconversion (RR 1.63 (95%CI 1.04, 2.57), but the association was weaker after correction for multiple testing. Furthermore, there was no evidence of association between I-FABP and rotavirus vaccine seropositivity or GMT (Tables S4 & S5). In adjusted analyses, log-rise in myeloperoxidase was positively associated with RVV seroconversion (RR 1.29 (95%CI 1.01, 1.65) (Table 1) and with both secondary outcomes (Tables S4 & S5). Again, these associations were weaker after *P* value adjustment. There were no other associations between individual EED biomarkers and rotavirus immunogenicity (Fig. 2) (Tables 1, S4 and S5).

**Table 1:** Associations between individual EED biomarkers and RVV seroconversion (primary outcome) among infants with specimens collected before RVV receipt.

Biomarker	Non-seroconverter			Seroconverter			Unadjusted analysis			Adjusted analysis		
	*N*	Mean	95% CI	*N*	Mean	95% CI	Unadj RR*	95% CI	*P* value	Adj RR*	95% CI	*P* value
**AAT** mg/mL	135	0.50	0.41, 0.63	46	0.59	0.39, 0.90	1.07	0.87, 1.30	0.540	1.07	0.87, 1.31	0.525
**CRP** mg/L	225	0.20	0.15, 0.25	73	0.16	0.11, 0.23	0.95	0.84, 1.07	0.374	0.95	0.84, 1.06	0.355
**CIT** ng/mL	218	2936	2813, 3064	73	2809	2635, 2995	0.76	0.44, 1.34	0.344	0.68	0.39, 1.21	0.189
**I-FABP** pg/mL	225	1338	1262, 1418	73	1517	1367, 1683	1.70	1.06, 2.73	0.027	1.63	1.04, 2.57	0.034
**KT** Ratio × 1000	189	52.7	50.7, 54.9	65	55.2	51.5, 59.2	1.40	0.70, 2.80	0.348	1.32	0.65, 2.70	0.442
**MPO** ng/mL	137	5160	4316, 6170	46	6559	4868, 8838	1.17	0.93, 1.47	0.174	1.29	1.01, 1.65	0.039
**NEO** nmol/L	137	818	742, 902	45	751	623, 907	0.80	0.52, 1.23	0.309	0.87	0.58, 1.34	0.538
**REG1B** ug/mL	133	16.1	13.3, 19.4	43	14.7	10.4, 20.8	0.96	0.77, 1.20	0.736	1.06	0.86, 1.32	0.578
**sCD14** pg/mL	225	6.5 × 10^5	6.0 × 10^5, 7.0 × 10^5	73	6.5 × 10^5	5.7 × 10^5, 7.5 x10^5	1.04	0.74, 1.46	0.825	1.12	0.79, 1.57	0.529

Geometric means are shown for biomarker values. Models were adjusted for WASH arm, season of birth, breastfeeding status and weight-for-age *Z*-score around the time of vaccination. *P* values highlighted in **bold** remained significant after adjusting for multiple testing using the Benjamini-Hochberg procedure.

* Risk ratio corresponding to an increase of one natural log of the biomarker.

### 3.4 Domains and scores of EED

There were no meaningful associations between EED domains (intestinal permeability, intestinal damage, intestinal inflammation and systemic inflammation) and any measure of RVV immunogenicity (Fig. 2) (Tables S6, S7 & S8). Among 146 infants with all 9 biomarkers, four principal components explained 65% of the variance in the biomarker data (Fig. S2). The first component clearly grouped markers of intestinal inflammation (PC1). The loadings for PC2 to PC4 fell less clearly within our pre-specified domains but broadly combined markers of systemic inflammation (PC2), markers of intestinal epithelial health (PC3), and markers of gut damage, translocation and systemic inflammation (PC4). There were no associations between each principal component and any measure of RVV immunogenicity (Fig. 2) (Tables S6 & S8). Similarly, there were no associations between either of the EED scores and RVV immunogenicity. Mean (SD) cumulative tertile scores of all 9 biomarkers (SHINE score) were 8.5 among non-seroconverters versus 9.1 among seroconverters (aRR 1.07, 95% CI 0.96, 1.20) (Fig. 2) (Tables S6 & S8). Internal consistency of these composite scores, measured using McDonald's omega, was marginally better for the nine biomarker SHINE score than for the three biomarker Kosek score, 0.63 (95%CI 0.50, 0.69) versus 0.41 (95%CI 0.13, 0.52).

### 3.5 Sensitivity analysis

When associations between individual biomarkers and RVV immunogenicity were tested among a larger group of infants, with biomarkers measured before or after RVV receipt, inferences remained similar (Table S9).

### 3.6 WASH effects on EED measured around the time of RVV receipt

The effects of WASH on EED biomarkers was estimated in 505 infants with both RVV immunogenicity data and a valid EED biomarker measured in the first six months of life (199 WASH versus 306 non-WASH infants). Baseline characteristics were similar between WASH and non-WASH groups (Table S10). Overall, there was no meaningful impact of the randomised WASH intervention on any individual EED biomarker (Table 2).

**Table 2:** Concentrations of EED biomarkers among infants in the WASH and non-WASH groups.

Biomarker	Study Arm	*N*	GM (95% CI)	GM Ratio (95% CI)	*P* value
AAT (mg/mL)	Non-WASH	203	0.44 (0.35, 0.56)	ref	Ref
AAT (mg/mL)	WASH	120	0.41 (0.29, 0.60)	0.93 (0.61, 1.42)	0.773
CRP (mg/L)	Non-WASH	306	0.39 (0.30, 0.49)	ref	ref
CRP (mg/L)	WASH	199	0.45 (0.33, 0.62)	1.13 (0.74, 1.72)	0.533
Citrulline (ng/mL)	Non-WASH	298	2837 (2711, 2969)	ref	ref
Citrulline (ng/mL)	WASH	191	2752 (2625, 2886)	0.97 (0.91, 1.04)	0.352
IFABP (pg/mL)	Non-WASH	306	1264 (1192, 1340)	ref	ref
IFABP (pg/mL)	WASH	199	1176 (1091, 1267)	0.93 (0.85, 1.02)	0.136
KTR (ratio x 1000)	Non-WASH	266	52.9 (50.9, 55.0)	ref	ref
KTR (ratio x 1000)	WASH	168	51.2 (48.7, 53.8)	0.97 (0.91, 1.03)	0.370
MPO (ng/mL)	Non-WASH	205	2895 (1499, 5592)	ref	ref
MPO (ng/mL)	WASH	123	1324 (414, 4233)	0.44 (0.12, 1.58)	0.322
NEO (nmol/L)	Non-WASH	202	803 (701, 919)	ref	ref
NEO (nmol/L)	WASH	122	867 (705, 1067)	1.08 (0.85, 1.37)	0.571
REG1B (ug/mL)	Non-WASH	189	17.0 (14.3, 20.3)	ref	ref
REG1B (ug/mL)	WASH	112	16.6 (13.5, 20.5)	1.04 (0.66, 1.65)	0.848
sCD14 (ng/mL)	Non-WASH	306	705,605 (651,270, 764,474)	ref	ref
sCD14 (ng/mL)	WASH	199	721,223 (654,106, 795,227)	1.02 (0.9, 1.16)	0.744

AAT = alpha-1 antitrypsin, CRP = C-reactive protein, CIT = citrulline, GM = geometric mean, IFABP = intestinal fatty acid binding protein, KTR = kynurenine tryptophan ratio, MPO = myeloperoxidase, NEO = neopterin, REG1B = regenerating enzyme 1B, sCD14 = soluble CD14.

## 4 Discussion

In this study, we measured multiple biomarkers of EED among rural Zimbabwean infants but we did not find associations between elevated EED biomarkers and impaired rotavirus vaccine immunogenicity. If anything, infants with elevated I-FABP or myeloperoxidase were more likely to respond to vaccination, although these associations were weakened after adjustment for multiple comparisons. Our SHINE EED score, based on nine biomarkers, showed better internal consistency than a three biomarker score previously defined by Kosek and colleagues [10]. Nevertheless, this score and others grouping together multiple EED biomarkers were also not associated with RVV immunogenicity. Collectively, we interpret these results as showing no evidence – using currently available biomarkers – of an inhibitory effect of EED on oral rotavirus vaccine immunogenicity.

Two previous studies similarly found a positive association between I-FABP concentration and oral vaccine responses. The first, in 40 Bangladeshi children aged 3–14 years, found an association between I-FABP and cholera toxin-specific effector memory T-cell responses [32]. The second study measured anti-rotavirus IgA among 142 Zambian infants [33]; elevated I-FABP was associated with a small increase in RVV seroconversion (adjusted RR 1.07 (95% CI 1.02, 1.13)). However, neither study used false-discovery rate adjustment. I-FABP is found in mature enterocytes, particularly at the small intestinal villous tips, and is released into the circulation when the cell membrane is compromised [34]. It is a dynamic measure of acute enterocyte damage, but may be less informative of chronic injury [35], particularly in low-income countries [36,37]. It is plausible that intestinal epithelial injury actually enhances immune responses to oral vaccines, by influencing entry into enterocytes or virus replication. Investigators have suggested that enteric pathogens, which are associated with intestinal injury, can behave as immune stimulators [38]. Elevated I-FABP, resulting from pathogen-associated damage, may partly reflect this process.

A similar explanation may underlie the positive association between myeloperoxidase and RVV immunogenicity. Stool myeloperoxidase is a marker of neutrophil recruitment to intestinal tissues. Neutrophil proteases, which are involved in bacterial killing, may in fact facilitate uptake of a viral vaccine. For membrane penetration, the outer capsid VP4 spike protein on the rotavirus surface must be proteolytically cleaved into two fragments by trypsin-like proteases [39]. However, much remains unknown about the cell surface molecules that serve as functional receptors for rotaviruses [40], or the implications of cell surface damage. Moreover, the evidence supporting an enhancing effect of intestinal inflammation on vaccine response is extremely heterogeneous. Among 590 Bangladeshi infants aged 3 months in the PROVIDE study, increased stool calprotectin (another marker of neutrophil activity) was associated with successful oral polio vaccine type 3 (OPV3) seroconversion [34], while there were no significant associations between myeloperoxidase and either OPV or RVV responses [34]. In an Indian trial of azithromycin versus placebo for 3 days prior to receiving serotype-3 monovalent OPV, there were no associations between myeloperoxidase and OPV3 response among 6–11 month old children [41]. In contrast, in a study of Nicaraguan infants, elevated myeloperoxidase was associated with failure to seroconvert to Rotateq^TM^ [11].

KT ratio measures the activity of IDO1, an immune checkpoint molecule expressed in immune and epithelial cells, and is therefore a composite marker of mucosal health and systemic inflammation [42]. IDO1 is the rate-limiting enzyme in the conversion of tryptophan to kynurenine; therefore low tryptophan, high kynurenine and high KTR indicate increased IDO1 activity. KTR has recently been used as an EED biomarker in other studies [43,44] but there are no published data to our knowledge exploring its relationship with RVV immunogenicity. Among Peruvian infants, elevated KTR was associated with increased odds of OPV1 failure (OR 1.89 (95% CI 1.21, 2.97)), driven by a relative increase in kynurenine [43]. By contrast, in our analysis, KTR was not associated with RVV seroconversion. Prior studies suggest that systemic inflammation does not interfere with rotavirus vaccine responses in Africa. Among HIV-infected infants with heightened pro-inflammatory cytokines and monocyte activation, IgA responses to Rotateq^TM^ were equivalent to responses in HIV-uninfected infants with no inflammation [45,46].

We previously reported that household improvements in WASH led to increased rotavirus immunogenicity [13]. However, we found no evidence of WASH effects on biomarkers of EED around the time of vaccination, so these measures of EED are unlikely to explain our previous results. This is consistent with the findings from a larger study using the same population of infants, which explored the evolution of EED biomarkers from birth to 18 months and the effects of the SHINE trial interventions on EED at several time points [47].

This study had several strengths. We evaluated an extensive panel of gut biomarkers in infants, which may be more sensitive to early changes in intestinal health than direct visualisation of the small intestine [7]. Furthermore, this is the first study to explore relationships between citrulline, KT ratio, and rotavirus vaccine immunogenicity. Nevertheless, there are some important limitations to this analysis. Firstly, low rates of seroconversion, coupled with a relatively small number of infants with pre-vaccine samples, reduced our power to detect meaningful associations. Secondly, urinary lactulose-mannitol ratios were not measured at the 1-month visit (due to concerns about interrupting exclusive breastfeeding with oral dosing of lactulose and mannitol)*,* so assessment of intestinal permeability was reliant on a single biomarker (AAT). This limits our ability to draw comparisons with other studies, although we note that this dual sugar absorption test is prone to significant variability between studies in dosing, methods of analysis and interpretation [48]. Finally, the definition of EED remains problematic. It remains unclear what pathological processes each biomarker captures, how biomarkers relate to one another, and what reference ranges should be used in low-income countries. Results from the RoVI (*Ro*tavirus *V*accine *I*mmunogenicity) study, which measured EED biomarkers in the UK, India and Malawi and is exploring associations with RVV immunogenicity, will hopefully shed more light on this [49].

Overall, our findings indicate elevated EED biomarkers in Zimbabwean infants soon after birth, but no deleterious impact on oral rotavirus vaccine responses. We found modest associations between both elevated I-FABP and myeloperoxidase and increased rotavirus vaccine immunogenicity; however, these results require further mechanistic exploration and should be interpreted with caution given the heterogeneous findings in other studies. Interventions targeting EED may not be effective in improving oral RVV performance, and further studies are needed to understand the impact of the intestinal milieu on oral vaccine immunogenicity.

## Contributors

JAC, JHH and AJP designed the sub-study. BL, MPC, MM, JRS and BDK developed the laboratory techniques. JAC, MG, SR, KM, GP and NG performed the laboratory assays. NVT managed field operations and FDM supervised all data collection in the trial. JAC, BC, RN, LM, EG and AJP accessed and were responsible for the raw data associated with the study. JAC, BC, RN, LM and EG and AJP developed the statistical models, and analyzed and interpreted the data. JAC wrote the first draft of the manuscript. All authors were involved in subsequent revisions of the manuscript for important intellectual content and have read and approved this final version to be published.

## Funding

This work was supported by the Wellcome Trust. The SHINE trial was funded by the Bill & Melinda Gates Foundation; UK Department for International Development (UK Aid); Swiss Agency for Development and Cooperation and US National Institutes of Health.

## Data sharing statement

The datasets generated during and/or analysed during the current study are available from the corresponding author on reasonable request.

## Declaration of Competing Interest

The authors whose names are listed above certify that they have NO affiliations or conflicts of interest that could have appeared to influence the work reported in this paper.

## Appendix Supplementary materials

Image, application 1

Image, application 2

## References

[1] Glass R.I., Parashar U.D., Bresee J.S. (2006). Rotavirus vaccines: current prospects and future challenges. Lancet.

[2] Madhi S.A., Cunliffe N.A., Steele D. (2010). Effect of human rotavirus vaccine on severe diarrhea in African infants. N Engl J Med.

[3] Ruiz-Palacios G.M., Perez-Schael I., Velazquez F.R. (2006). Safety and efficacy of an attenuated vaccine against severe rotavirus gastroenteritis. N Engl J Med.

[4] Patel M., Shane A.L., Parashar U.D. (2009). Oral rotavirus vaccines: how well will they work where they are needed most?. J Infect Dis.

[5] Parker E.P., Ramani S., Lopman B.A. (2018). Causes of impaired oral vaccine efficacy in developing countries. Future Microbiol.

[6] Kelly P., Menzies I., Crane R. (2004). Responses of small intestinal architecture and function over time to environmental factors in a tropical population. Am J Trop Med Hyg.

[7] Keusch G.T., Denno D.M., Black R.E. (2014). Environmental enteric dysfunction: pathogenesis, diagnosis, and clinical consequences. Clin Infect Dis.

[8] Prendergast A., Kelly P. (2012). Enteropathies in the developing world: neglected effects on global health. Am J Trop Med Hyg.

[9] Cheng W.D., Wold K.J., Bollinger L.B. (2019). Supplementation with lactoferrin and lysozyme ameliorates environmental enteric dysfunction: a double-blind, randomized, placebo-controlled trial. Am J Gastroenterol.

[10] Kosek M., Haque R., Lima A. (2013). Fecal markers of intestinal inflammation and permeability associated with the subsequent acquisition of linear growth deficits in infants. Am J Trop Med Hyg.

[11] Becker-Dreps S., Vilchez S., Bucardo F. (2017). The association between fecal biomarkers of environmental enteropathy and rotavirus vaccine response in Nicaraguan infants. Pediatr Infect Dis J.

[12] Church J.A., Parker E.P., Kosek M.N. (2018). Exploring the relationship between environmental enteric dysfunction and oral vaccine responses. Future Microbiol.

[13] Church J.A., Rukobo S., Govha M. (2019). The impact of improved water, sanitation and hygiene on oral rotavirus vaccine immunogenicity in Zimbabwean infants: sub-study of a cluster-randomized trial. Clin Infect Dis.

[14] Humphrey J.H., Jones A.D., Manges A. (2015). The sanitation hygiene infant nutrition efficacy (SHINE) trial: rationale. Des Methods Clin Infect Dis.

[15] Humphrey J.H., Mbuya M.N.N., Ntozini R. (2019). Independent and combined effects of improved water, sanitation, and hygiene, and improved complementary feeding, on child stunting and anaemia in rural Zimbabwe: a cluster-randomised trial. Lancet Glob Health.

[16] UNICEF, Zimbabwe: WHO and UNICEF estimates of immunization coverage: 2016 revision. 2016.

[17] Prendergast A.J., Humphrey J.H., Mutasa K. (2015). Assessment of environmental enteric dysfunction in the SHINE trial: methods and challenges. Clin Infect Dis.

[18] GlaxoSmithKline. Prescribing information (ROTARIX). 2019 18 July 2019]; Available from: https://www.gsksource.com/pharma/content/dam/GlaxoSmithKline/US/en/Prescribing_Information/Rotarix/pdf/ROTARIX-PI-PIL.PDF.

[19] Patel M., Glass R.I., Jiang B. (2013). A systematic review of anti-rotavirus serum IgA antibody titer as a potential correlate of rotavirus vaccine efficacy. J Infect Dis.

[20] Bernstein D.I., Smith V.E., Sherwood J.R. (1998). Safety and immunogenicity of live, attenuated human rotavirus vaccine 89-12. Vaccine.

[21] Ward R.L., Bernstein D.I., Shukla R. (1989). Effects of antibody to rotavirus on protection of adults challenged with a human rotavirus. J Infect Dis.

[22] Kaiser H.F. (1960). The application of electronic computers to factor analysis. Educ Psychol Meas.

[23] Hastings C., Mosteller F., Tukey J.W. (1947). Low moments for small samples: a comparative study of order statistics. Ann Math Stat.

[24] Benjamini Y., Hochberg Y. (1995). Controlling the false discovery rate: a practical and powerful approach to multiple testing. J R Stat Soc Ser B Methodol.

[25] Kleiber, C. and A. Zeileis, AER: applied econometrics with R. 2016, R package version 1.2-5. p. https://cran.r-project.org/web/packages/AER/index.html.

[26] Berger S., Graham N., Zeileis A. (2017).

[27] Højsgaard, S., U. Halekoh, and J. Yan, The R package Geepack for generalized estimating equations. 2005, 2005. 15(2): p. 11.

[28] Ledjeff E., Artner-Dworzak E., Witasek A. (2001).

[29] Lodrup Carlsen K.C., Lovik M., Granum B. (2006). Soluble CD14 at 2yr of age: gender-related effects of tobacco smoke exposure, recurrent infections and atopic diseases. Pediatr Allergy Immunol.

[30] Meyers S., Wolke A., Field S.P. (1985). Fecal alpha 1-antitrypsin measurement: an indicator of Crohn's disease activity. Gastroenterology.

[31] Saiki T. (1998). Myeloperoxidase concentrations in the stool as a new parameter of inflammatory bowel disease. Kurume Med J.

[32] Uddin M.I., Islam S., Nishat N.S. (2016). Biomarkers of environmental enteropathy are positively associated with immune responses to an oral cholera vaccine in Bangladeshi children. PLoS Negl Trop Dis.

[33] Mwape I., Bosomprah S., Mwaba J. (2017). Immunogenicity of rotavirus vaccine (RotarixTM) in infants with environmental enteric dysfunction. PLoS ONE.

[34] Naylor C., Lu M., Haque R. (2015). Environmental enteropathy, oral vaccine failure and growth faltering in infants in Bangladesh. EBioMedicine.

[35] Peoc'h K., Nuzzo A., Guedj K. (2018). Diagnosis biomarkers in acute intestinal ischemic injury: so close, yet so far. Clin Chem Lab Med.

[36] Olwenyi O.A., Naluyima P., Cham F. (2016). Brief report: differential associations of interleukin 6 and intestinal fatty acid-binding protein with progressive untreated HIV-1 infection in Rakai, Uganda. J Acquir Immune Defic Syndr.

[37] Prendergast A.J., Chasekwa B., Rukobo S. (2017). Intestinal damage and inflammatory biomarkers in human immunodeficiency virus (HIV)-exposed and HIV-infected Zimbabwean infants. J Infect Dis.

[38] Harris V., Armah G., Fuentes S. (2017). Significant correlation between the infant gut microbiome and rotavirus vaccine response in rural Ghana. J Infect Dis.

[39] Glass R.I., Bresee J., Jiang B. (2006). Rotavirus and rotavirus vaccines. Adv Exp Med Biol.

[40] Mendez E., Arias C.F., Lopez S. (1993). Binding to sialic acids is not an essential step for the entry of animal rotaviruses to epithelial cells in culture. J Virol.

[41] Grassly N.C., Praharaj I., Babji S. (2016). The effect of azithromycin on the immunogenicity of oral poliovirus vaccine: a double-blind randomised placebo-controlled trial in seronegative Indian infants. Lancet Infect Dis.

[42] Richard D.M., Dawes M.A., Mathias C.W. (2009). l-Tryptophan: basic metabolic functions, behavioral research and therapeutic indications. Int J Tryptophan Res.

[43] Kosek M.N., Mduma E., Kosek P.S. (2016). Plasma tryptophan and the kynurenine-tryptophan ratio are associated with the acquisition of statural growth deficits and Oral vaccine underperformance in populations with environmental enteropathy. Am J Trop Med Hyg.

[44] Semba R.D., Trehan I., Li X. (2017). Environmental enteric dysfunction is associated with carnitine deficiency and altered fatty acid oxidation. EBioMedicine.

[45] Uprety P., Lindsey J.C., Levin M.J. (2017). Inflammation and immune activation in antiretroviral-treated human immunodeficiency virus type 1-infected African infants and rotavirus vaccine responses. J Infect Dis.

[46] Levin M.J., Lindsey J.C., Kaplan S.S. (2017). Safety and immunogenicity of a live attenuated pentavalent rotavirus vaccine in HIV-exposed infants with or without HIV infection in Africa. AIDS.

[47] Gough E.K., Moulton L.H., Mutasa K. (2020). Effects of improved water, sanitation, and hygiene and improved complementary feeding on environmental enteric dysfunction in children in rural Zimbabwe: a cluster-randomized controlled trial. PLoS Negl Trop Dis.

[48] Denno D.M., VanBuskirk K., Nelson Z.C. (2014). Use of the lactulose to mannitol ratio to evaluate childhood environmental enteric dysfunction: a systematic review. Clin Infect Dis.

[49] Sindhu K.N., Cunliffe N., Peak M. (2017). Impact of maternal antibodies and infant gut microbiota on the immunogenicity of rotavirus vaccines in African, Indian and European infants: protocol for a prospective cohort study. BMJ Open.

