# Supplementary material for: Associations between biomarkers of environmental enteric dysfunction and oral rotavirus vaccine immunogenicity in rural Zimbabwean infants
Source: eClinicalMedicine. 2021 Nov 15;41:101173. doi: 10.1016/j.eclinm.2021.101173 (PMC8605235; doi:10.1016/j.eclinm.2021.101173)
Supplement: Supplementary file 1 [file mmc1.docx]

**Captions for supplementary material**

*All our supplementary tables and figures can be found together in a single supplementary file* ***210328_RV_EED_appendix****.doc, which can be referred to as “appendix” in the main manuscript text.*
