# Supplementary material for: Associations between biomarkers of environmental enteric dysfunction and oral rotavirus vaccine immunogenicity in rural Zimbabwean infants
Source: eClinicalMedicine. 2021 Nov 15;41:101173. doi: 10.1016/j.eclinm.2021.101173 (PMC8605235; doi:10.1016/j.eclinm.2021.101173)
Supplement: Supplementary file 2 [file mmc2.docx]

**Associations between biomarkers of environmental enteric dysfunction and oral rotavirus vaccine immunogenicity in rural Zimbabwean infants**

James A Church^1,2 #^, Sandra Rukobo^1^, Margaret Govha^1^, Ethan K Gough^1,6^, Bernard Chasekwa^1^, Benjamin Lee^3^, Marya P Carmolli^4^, Gordana Panic^5^, Natasa Giallourou^5^, Robert Ntozini^1^, Kuda Mutasa^1^, Monica M McNeal^6^, Florence D. Majo^1^, Naume V. Tavengwa^1^, Jonathan R. Swann^5^, Lawrence H Moulton^7^, Beth D Kirkpatrick^4^, Jean H Humphrey^1,7^, Andrew J Prendergast^1,2,7 $^

**Table of Contents for Appendix**

| **Supplementary tables** |  |
| --- | --- |
| 1. Table S1: Biomarker domains and domain scoring system | Page 2 |
| 1. Table S2: Baseline characteristics of infants & mothers enrolled in each analysis. | Page 3 |
| 1. Table S3a: Baseline characteristics (RVV seroconverters vs non-seroconverters) | Page 4 |
| 1. Table S3b: Baseline characteristics (complete EED data vs incomplete EED data) | Page 5 |
| 1. Table S4: Associations between individual EED biomarkers and RVV seropositivity | Page 6 |
| 1. Table S5. Associations between individual EED biomarkers and RVV GMT | Page 7 |
| 1. Table S6. Associations between EED biomarker scores and RVV seroconversion | Page 8 |
| 1. Table S7. Associations between EED biomarker domains and RVV seroconversion | Page 9 |
| 1. Table S8. Associations between EED biomarker scores and secondary outcomes | Page 10 |
| 1. Table S9. Sensitivity analysis (EED vs immunogenicity in larger population) | Page 11 |
| 1. Table S10. Baseline characteristics (WASH vs non-WASH) | Page 13 |
| **Supplementary figures** |  |
| 1. Figure S1: Burden of EED among infants | Page 14 |
| 1. Figure S2: Scree plot and table showing principal components | Page 15 |

**Supplementary tables**

**Table S1.** EED biomarker domains and scoring system based on percentile categories

For each domain, composite scores were created using percentile categories from biomarkers in that domain, with categories defined as 0 (<25^th^ centile), 1 (25^th^-75^th^ percentile), or 2 (>75^th^ percentile). E.g. a child with MPO in the >75^th^ percentile and NEO in the 25^th^-75^th^ percentile would be assigned an intestinal inflammation domain score of 3 (2+1).

**Table S2.** Baseline^#^ characteristics of infants, mothers and their households enrolled in each analysis.

|  | **EED RVV analysis**  (Infants N=303) | **WASH EED analysis**  (Infants N=505) | **All HIV-unexposed**  (Infants N= 3484)* |
| --- | --- | --- | --- |
| **Infant characteristics**  Gender, % female  Birthweight, kilograms; mean (SD)  Low birthweight (<2.5kg), %  Institutional delivery, %  Normal Vaginal delivery, %  Born in rotavirus season, %  Exclusive breastfeeding (EBF), %  Receipt of concurrent OPV, % | 45.5  3.16 (0.5)  5.9  92.3  94.1  37.3  95.3  96.4 | 48.9  3.14 (0.5)  7.7  91.6  94.1  37.6  93.5  95.1 | 49.4  3.09 (0.53)  8.2  88.6  92.5  33.4  87.1  n/a |
| **Maternal characteristics**  Age, years; mean (SD)  Parity, median (IQR)  Height, cm; mean (SD)  MUAC, cm; mean (SD)  Married, %  Completed years of school, median (IQR)  Unemployed, %  Religion:  Apostolic, %  Other Christian, %  Other religion, %  Wealth Quintile:  Lowest, %  Second ,%  Middle, %  Fourth, %  Highest, % | 27.1 (6.0)  2 (2, 2)  160.5 (5.7)  27.1 (3.2)  96.6  10 (9, 11)  90.5  43.7  37.7  18.6  15.3  21.0  23.4  22.7  17.6 | 27.0 (6.4)  2 (2, 2)  160.2 (5.8)  26.9 (3.3)  95.4  10 (9, 11)  89.6  46.7  34.9  18.4  16.4  20.5  23.8  19.7  19.5 | 25.3 (7.7)  2 (1, 3)  160.1 (5.5)  26.3 (3.2)  95.5  10 (9,11)  92.0  47.4  44.2  18.4  17.4  19.1  18.8  21.0  19.3 |
| **Household characteristics**  Household size, median (IQR)  Electricity, %  Open defecation, %  Any latrine, %  Improved latrine, %  Improved water source, %  Handwashing station present, %  Improved floor, %  Owns chickens, %  Livestock observed in house, % | 5 (3, 6)  2.7  48.6  39.9  33.6  66.9  7.9  57.6  82.0  47.2 | 5 (4, 6)  2.5  47.6  41.2  36.2  67.2  11.4  53.2  81.4  45.4 | 5 (3,6)   2.7  48.4  40.0  35.2  63.1  8.4  55.4  83.1  35.9 |

^#^ Baseline for infants was at birth (except EBF, which was determined at the visit closest to the first dose of RVV); baseline for maternal and household characteristics was two weeks after consent (~14 weeks gestation). ^*^ All live born HIV-unexposed infants in SHINE who were not enrolled for the WASH EED analysis i.e. 3989 minus 505.

MUAC = mid-upper arm circumference; n/a = data not available

**Table S3a.** Baseline^#^ characteristics of infants, mothers and their households compared between RVV seroconverters and RVV non-seroconverters.

|  | **RVV non-seroconverters**  (Infants N=224) | **RVV seroconverters**  (Infants N=79) |
| --- | --- | --- |
| **Infant characteristics**  Gender, % female  Birthweight, kilograms; mean (SD)  Low birthweight (<2.5kg), %  Institutional delivery, %  Normal Vaginal delivery, %  Born in rotavirus season, %  Exclusive breastfeeding (EBF), %  WASH trial arm, % | 42.9  3.16 (0.5)  5.4  92.7  92.9  34.8  95.0  29.5 | 53.2  3.15 (0.5)  7.6  91.1  97.5  44.3  96.2  40.5 |
| **Maternal characteristics**  Age, years; mean (SD)  Parity, median (IQR)  Height, cm; mean (SD)  MUAC, cm; mean (SD)  Married, %  Completed years of school, median (IQR)  Unemployed, %  Religion:  Apostolic, %  Other Christian, %  Other religion, %  Wealth Quintile:  Lowest, %  Second ,%  Middle, %  Fourth, %  Highest, % | 27.4 (6.3)  2 (2, 2)  160.4 (5.6)  27.1 (3.2)  95.9  10 (9, 11)  92.2  44.1  36.7  19.2  13.4  21.7  22.6  23.0  19.4 | 26.3 (5.2)  2 (2, 2)  161.0 (6.0)  27.2 (3.3)  98.7  10 (9, 11)  85.9  42.3  38.4  19.3  20.5  19.2  25.6  21.8  12.8 |
| **Household characteristics**  Household size, median (IQR)  Electricity, %  Open defecation, %  Any latrine, %  Improved latrine, %  Improved water source, %  Handwashing station present, %  Improved floor, %  Owns chickens, %  Livestock observed in house, % | 5 (4, 6)  2.8  46.5  41.0  34.4  71.1  6.4  61.0  83.0  46.6 | 5 (3, 6)  2.6  54.5  36.5  31.1  55.3  11.8  48.1  79.5  48.7 |

^#^ Baseline for infants was at birth (except EBF, which was determined at the visit closest to the first dose of RVV); baseline for maternal and household characteristics was two weeks after consent (~14 weeks gestation).

**Table S3b.** Baseline^#^ characteristics compared between infants with complete EED biomarker data and infants with incomplete EED biomarker data.

|  | **Incomplete**  **EED biomarker data**  (Infants N=157) | **Complete**  **EED biomarker data**  (Infants N=146) |
| --- | --- | --- |
| **Infant characteristics**  Gender, % female  Birthweight, kilograms; mean (SD)  Low birthweight (<2.5kg), %  Institutional delivery, %  Normal Vaginal delivery, %  Born in rotavirus season, %  Exclusive breastfeeding (EBF), %  WASH trial arm, % | 52.9  3.15 (0.5)  7.0  89.6  95.5  33.8  97.4  33.1 | 37.7  3.16 (0.4)  4.8  95.2  92.5  41.1  93.1  31.5 |
| **Maternal characteristics**  Age, years; mean (SD)  Parity, median (IQR)  Height, cm; mean (SD)  MUAC, cm; mean (SD)  Married, %  Completed years of school, median (IQR)  Unemployed, %  Religion:  Apostolic, %  Other Christian, %  Other religion, %  Wealth Quintile:  Lowest, %  Second ,%  Middle, %  Fourth, %  Highest, % | 26.9 (5.9)  2 (2, 2)  160.0 (6.0)  26.8 (3.4)  95.4  10 (8, 11)  92.0  46.8  40.4  12.8  18.0  24.0  21.3  20.0  16.7 | 27.4 (6.2)  2 (2, 2)  161.2 (5.4)  27.4 (3.0)  97.9  10 (9, 11)  89.0  40.4  39.1  20.5  12.4  17.9  25.5  25.5  18.6 |
| **Household characteristics**  Household size, median (IQR)  Electricity, %  Open defecation, %  Any latrine, %  Improved latrine, %  Improved water source, %  Handwashing station present, %  Improved floor, %  Owns chickens, %  Livestock observed in house, % | 5 (3, 6)  3.3  52.0  33.8  28.9  66.4  5.5  51.7  80.7  49.7 | 5 (4, 6)  2.1  45.1  45.8  38.2  67.4  10.4  63.8  83.5  44.5 |

^#^ Baseline for infants was at birth (except EBF, which was determined at the visit closest to the first dose of RVV); baseline for maternal and household characteristics was two weeks after consent (~14 weeks gestation).

Table S4. Associations between individual EED biomarkers and RVV seropositivity (secondary outcome) among infants with specimens collected before RVV receipt.

| **Biomarker** | **Seronegative** | | | **Seropositive** | | | Unadjusted analysis | | | Adjusted analysis ^#^ | | |
| --- | --- | --- | --- | --- | --- | --- | --- | --- | --- | --- | --- | --- |
|  | **N** | **Mean ^&^** | **95% CI** | **N** | **Mean ^&^** | **95% CI** | **Unadj RR ^*^** | **95% CI** | **P value** | **Adj RR ^*^** | **95% CI** | **P value** |
| **AAT**  mg/mL | 134 | 0.49 | 0.39, 0.60 | 51 | 0.58 | 0.38, 0.87 | 1.06 | 0.88, 1.28 | 0.521 | 1.07 | 0.89, 1.29 | 0.481 |
| **CRP**  mg/L | 221 | 0.19 | 0.15, 0.24 | 79 | 0.18 | 0.12, 0.25 | 0.98 | 0.87, 1.09 | 0.676 | 0.97 | 0.87, 1.09 | 0.618 |
| **CIT**  ng/mL | 214 | 2921 | 2798, 3050 | 79 | 2868 | 2694, 3053 | 0.97 | 0.55, 1.69 | 0.904 | 0.90 | 0.50, 1.62 | 0.734 |
| **IFABP**  pg/mL | 221 | 1346 | 1270, 1426 | 79 | 1478 | 1335, 1636 | 1.50 | 0.97, 2.31 | 0.068 | 1.43 | 0.95, 2.18 | 0.088 |
| **KT**  ratio × 1000 | 185 | 52.8 | 50.7, 55.0 | 71 | 54.6 | 51.0, 58.5 | 1.22 | 0.63, 2.39 | 0.548 | 1.13 | 0.56, 2.26 | 0.730 |
| **MPO**  ng/mL | 136 | 5132 | 4284, 6148 | 51 | 6793 | 5131, 8995 | 1.20 | 0.98, 1.48 | 0.082 | 1.29 | 1.02, 1.63 | 0.031 |
| **NEO**  nmol/L | 136 | 804 | 728, 889 | 50 | 743 | 624, 884 | 0.83 | 0.56, 1.21 | 0.331 | 0.88 | 0.59, 1.30 | 0.514 |
| **REG1B**  ug/mL | 131 | 15.9 | 13.2, 19.2 | 47 | 15.5 | 11.2, 21.6 | 0.99 | 0.81, 1.22 | 0.981 | 1.08 | 0.88, 1.33 | 0.448 |
| **sCD14**  pg/mL | 225 | 6.4x10^5 | 5.9 x10^5, 7.0x10^5 | 79 | 6.8x10^5 | 6.0 x10^5, 7.8 x10^5 | 1.17 | 0.84, 1.63 | 0.361 | 1.26 | 0.90, 1.77 | 0.173 |

AAT = alpha-1 antitrypsin, CRP = C-reactive protein, CIT = citrulline, IFABP = intestinal fatty acid binding protein, KTR = kynurenine tryptophan ratio, MPO = myeloperoxidase, NEO = neopterin, REG1B = regenerating enzyme 1B, sCD14 = soluble CD14.

* Risk ratio corresponding to a unit increase (one natural log) in the biomarker.

**^&^** Geometric means are shown for biomarker values.

^#^ Models were adjusted for WASH arm, season of birth, breastfeeding status and weight-for-age Z-score around the time of vaccination.

P values highlighted in **bold** remained significant after adjusting for multiple testing using the Benjamini-Hochberg procedure.

Table S4. Associations between individual EED biomarkers and RVV GMT (secondary outcome) among infants with specimens collected before RVV receipt.

| Biomarker | Rotavirus vaccine GMT | | | | | |
| --- | --- | --- | --- | --- | --- | --- |
|  | **Unadj**  **GMT ratio ^*^** | **95% CI** | **P value** | **Adj ^#^**  **GMT Ratio ^*^** | **95% CI** | **P value** |
| AAT  mg/mL | 1.17 | 0.73, 1.88 | 0.512 | 1.21 | 0.75, 1.93 | 0.433 |
| CRP  mg/L | 0.86 | 0.65, 1.13 | 0.274 | 0.81 | 0.61, 1.08 | 0.155 |
| CIT  ng/mL | 0.73 | 0.15, 3.54 | 0.699 | 0.75 | 0.15, 3.81 | 0.729 |
| IFABP  pg/mL | 2.39 | 0.83, 6.87 | 0.105 | 2.43 | 0.81, 7.31 | 0.114 |
| KT  ratio | 3.21 | 0.53, 19.3 | 0.202 | 2.51 | 0.42, 14.9 | 0.312 |
| MPO  ng/mL | 1.80 | 0.98, 3.30 | 0.057 | 1.98 | 1.03, 3.82 | 0.039 |
| NEO  nmol/L | 0.61 | 0.21, 1.71 | 0.345 | 0.54 | 0.18, 1.63 | 0.276 |
| REG1B  ug/mL | 1.14 | 0.65, 2.00 | 0.638 | 1.35 | 0.76, 2.38 | 0.303 |
| sCD14  pg/mL | 1.64 | 0.73, 3.70 | 0.233 | 1.82 | 0.79, 4.18 | 0.157 |

AAT = alpha-1 antitrypsin, CRP = C-reactive protein, CIT = citrulline, IFABP = intestinal fatty acid binding protein, KTR = kynurenine tryptophan ratio, MPO = myeloperoxidase, NEO = neopterin, REG1B = regenerating enzyme 1B, sCD14 = soluble CD14.

* GMT ratio corresponding to a unit increase in the EED biomarker score.

^#^ Models were adjusted for WASH arm, season of birth, breastfeeding status and weight-for-age Z-score around the time of vaccination.

P values highlighted in **bold** remained significant after adjusting for multiple testing using the Benjamini-Hochberg procedure.

Table S5. Associations between EED biomarker scores (domains, principal component scores and total scores) and RVV seroconversion (primary outcome) among infants with specimens collected before RVV receipt.

| **Biomarker** | **Non-seroconverters** | | | **Seroconverters** | | | Unadjusted analysis | | | Adjusted analysis ^#^ | | |
| --- | --- | --- | --- | --- | --- | --- | --- | --- | --- | --- | --- | --- |
|  | **N** | **Mean** | **s.d.** | **N** | **Mean** | **s.d.** | **Unadj RR*** | **95% CI** | **P value** | **Adj RR*** | **95% CI** | **P value** |
| **Permeability (max=2)** | 135 | 0.99 | 0.70 | 46 | 1.09 | 0.72 | 1.16 | 0.79, 1.71 | 0.436 | 1.19 | 0.81, 1.75 | 0.379 |
| **Damage**  **(max=6)** | 129 | 2.88 | 1.37 | 43 | 2.79 | 1.23 | 1.00 | 0.84, 1.20 | 0.982 | 1.08 | 0.91, 1.28 | 0.394 |
| **Inflammation (max=4)** | 137 | 1.97 | 0.97 | 45 | 2.09 | 1.12 | 1.06 | 0.81, 1.38 | 0.673 | 1.13 | 0.86, 1.48 | 0.371 |
| **Translocation (max=6)** | 189 | 3.02 | 1.42 | 59 | 3.03 | 1.29 | 1.00 | 0.86, 1.15 | 0.960 | 1.00 | 0.86, 1.18 | 0.968 |
| **PC 1** | 107 | -0.06 | 1.51 | 38 | 0.17 | 1.16 | 1.10 | 0.85, 1.41 | 0.478 | 1.09 | 0.86, 1.37 | 0.493 |
| **PC 2** | 107 | 0.01 | 1.16 | 38 | -0.01 | 0.99 | 0.96 | 0.78, 1.17 | 0.670 | 0.99 | 0.78, 1.25 | 0.914 |
| **PC 3** | 107 | 0.01 | 1.01 | 38 | -0.04 | 1.06 | 0.97 | 0.77 1.22 | 0.804 | 0.95 | 0.76, 1.19 | 0.674 |
| **PC 4** | 107 | -0.05 | 1.05 | 38 | 0.15 | 0.83 | 0.97 | 0.76, 1.25 | 0.831 | 1.04 | 0.81, 1.33 | 0.786 |
| **EED SHINE (max=18)** | 107 | 8.55 | 2.56 | 38 | 9.11 | 2.61 | 1.06 | 0.96, 1.18 | 0.258 | 1.07 | 0.96, 1.20 | 0.246 |
| **EED Kosek (max=10)** | 135 | 4.90 | 2.42 | 45 | 5.38 | 2.62 | 1.06 | 0.95, 1.18 | 0.325 | 1.08 | 0.96, 1.20 | 0.205 |

PC = principal component.

* Risk ratio corresponding to a unit increase in the EED biomarker score.

^#^ Models were adjusted for WASH arm, season of birth, breastfeeding status and weight-for-age Z-score around the time of vaccination.

P values highlighted in **bold** remained significant after adjusting for multiple testing using the Benjamini-Hochberg procedure.

Table S6. Associations between EED biomarker domains (where *n =* any in top quartile) and RVV seroconversion (primary outcome) among infants with specimens collected before rotavirus vaccine receipt.

| **EED domain** | **Non-seroconverters** | | | **Seroconverters** | | | **Unadjusted analysis** | | | **Adjusted analysis ^#^** | | |
| --- | --- | --- | --- | --- | --- | --- | --- | --- | --- | --- | --- | --- |
|  | **N** | ***n*** | **%** | **N** | ***n*** | **%** | **Unadj RR^*^** | **95% CI** | **P value** | **Adj RR ^*^** | **95% CI** | **P value** |
| **Permeability** | 135 | 32 | 23.7 | 46 | 14 | 30.4 | 1.28 | 0.77, 2.14 | 0.337 | 1.27 | 0.76, 2.12 | 0.353 |
| **Damage** | 129 | 75 | 58.1 | 43 | 22 | 51.2 | 0.87 | 0.53, 1.41 | 0.567 | 0.94 | 0.58, 1.52 | 0.799 |
| **Inflammation** | 137 | 58 | 42.3 | 45 | 18 | 40.0 | 0.92 | 0.57, 1.48 | 0.727 | 1.04 | 0.65, 1.67 | 0.865 |
| **Translocation** | 189 | 104 | 55.0 | 65 | 34 | 52.3 | 0.89 | 0.58, 1.36 | 0.577 | 0.93 | 0.59, 1.48 | 0.760 |
| **EED all** | 107 | 93 | 86.9 | 38 | 34 | 89.5 | 1.28 | 0.55, 2.95 | 0.569 | 1.25 | 0.58, 2.71 | 0.563 |

* Risk ratio corresponding to a unit increase in the EED domain prevalence.

^#^ Models were adjusted for WASH arm, season of birth, breastfeeding status and weight-for-age Z-score around the time of vaccination.

P values highlighted in **bold** remained significant after adjusting for multiple testing using the Benjamini-Hochberg procedure.

Table S7. Associations between EED biomarker scores (domains, principal component scores and total scores) and both RVV seropositivity and GMT (secondary outcomes) among infants with specimens collected before rotavirus vaccine receipt.

| **EED score** | **Seronegative** | | | **Seropositive** | | | **Risk ratio^*^ for seropositivity** | | | | | | | **GMT ratio^*^** | | | | | |
| --- | --- | --- | --- | --- | --- | --- | --- | --- | --- | --- | --- | --- | --- | --- | --- | --- | --- | --- | --- |
|  | **N** | **Mean** | **s.d.** | **N** | **Mean** | **s.d.** | **Unadj RR** | **95% CI** | **P value** | **Adj^#^ RR** | **95% CI** | **P value** | **Unadj GMT ratio** | | **95% CI** | **P value** | **Adj^#^ GMT ratio** | **95% CI** | **P value** |
| **Permeability (max=2)** | 134 | 0.97 | 0.69 | 51 | 1.06 | 0.76 | 1.13 | 0.80, 1.61 | 0.492 | 1.15 | 0.80, 1.66 | 0.452 | 1.40 | | 0.60, 3.50 | 0.455 | 1.45 | 0.60, 3.50 | 0.406 |
| **Damage**  **(max=6)** | 126 | 2.88 | 1.37 | 47 | 2.83 | 1.24 | 1.02 | 0.86, 1.21 | 0.828 | 1.09 | 0.93, 1.28 | 0.288 | 1.07 | | 0.54, 1.64 | 0.808 | 1.13 | 0.69, 1.88 | 0.625 |
| **Inflammation (max=4)** | 136 | 1.96 | 0.97 | 50 | 2.08 | 1.14 | 1.08 | 0.85, 1.37 | 0.551 | 1.12 | 0.87, 1.44 | 0.363 | 1.28 | | 0.70, 2.33 | 0.417 | 1.24 | 0.65, 2.33 | 0.515 |
| **Translocation (max=6)** | 185 | 3.00 | 1.42 | 71 | 3.11 | 1.32 | 1.04 | 0.91, 1.18 | 0.593 | 1.04 | 0.90, 1.20 | 0.593 | 1.14 | | 0.79, 1.65 | 0.475 | 1.08 | 0.74, 1.57 | 0.690 |
| **PCA 1** | 104 | -0.09 | 1.52 | 42 | 0.22 | 1.14 | 1.15 | 0.88, 1.52 | 0.303 | 1.13 | 0.88, 1.45 | 0.343 | 1.45 | | 0.81, 2.61 | 0.215 | 1.39 | 0.79, 2.03 | 0.251 |
| **PCA 2** | 104 | -0.02 | 1.34 | 42 | -0.05 | 1.02 | 1.01 | 0.83, 1.23 | 0.952 | 1.03 | 0.84, 1.27 | 0.748 | 1.09 | | 0.60, 1.96 | 0.785 | 1.10 | 0.59, 1.86 | 0.771 |
| **PCA 3** | 104 | 0.03 | 1.14 | 42 | -0.09 | 1.04 | 0.92 | 0.74, 1.15 | 0.472 | 0.90 | 0.73, 1.12 | 0.343 | 0.75 | | 0.40, 1.43 | 0.383 | 0.71 | 0.38, 1.34 | 0.296 |
| **PCA 4** | 104 | 0.01 | 1.02 | 42 | -0.02 | 1.03 | 0.98 | 0.78, 1.23 | 0.876 | 1.04 | 0.84, 1.31 | 0.699 | 1.24 | | 0.62, 2.46 | 0.541 | 1.45 | 0.72, 2.75 | 0.316 |
| **EED SHINE (max=18)** | 104 | 8.46 | 2.53 | 42 | 9.29 | 2.59 | 1.10 | 0.99, 1.21 | 0.065 | 1.10 | 0.99, 1.23 | 0.068 | 1.30 | | 0.99, 1.72 | 0.059 | 1.29 | 0.99, 1.70 | 0.063 |
| **EED Kosek (max=10)** | 134 | 4.85 | 2.37 | 50 | 5.32 | 2.72 | 1.06 | 0.96, 1.17 | 0.289 | 1.06 | 0.96, 1.19 | 0.251 | 1.16 | | 0.90, 1.49 | 0.247 | 1.17 | 0.91, 1.51 | 0.218 |

PC = principal component.

* Risk ratio or GMT ratio corresponding to a unit increase in the EED biomarker score.

^#^ Models were adjusted for WASH arm, season of birth, breastfeeding status and weight-for-age Z-score around the time of vaccination.

P values highlighted in **bold** remained significant after adjusting for multiple testing using the Benjamini-Hochberg procedure.

Table S8. Associations between A) individual EED biomarkers and B) EED biomarker scores (domains and total scores) and RVV seropositivity among infants with specimens collected both before and after RVV receipt.

*Table S8-A*

| **Biomarker** | **Seronegative** | | | **Seropositive** | | | **Unadjusted analysis** | | |
| --- | --- | --- | --- | --- | --- | --- | --- | --- | --- |
|  | **N** | **Mean ^&^** | **95% CI** | **N** | **Mean ^&^** | **95% CI** | **Unadj RR^*^** | **95% CI** | **P value** |
| **AAT**  mg/mL | 220 | 0.51 | 0.43, 0.60 | 83 | 0.52 | 0.39, 0.68 | 1.01 | 0.86, 1.18 | 0.900 |
| **CRP**  mg/L | 380 | 0.42 | 0.34, 0.52 | 125 | 0.32 | 0.23, 0.45 | 0.96 | 0.89, 1.03 | 0.253 |
| **CIT**  ng/mL | 365 | 2816 | 2722, 2913 | 124 | 2829 | 2682, 2983 | 1.05 | 0.70, 1.58 | 0.820 |
| **IFABP**  pg/mL | 380 | 1203 | 1150, 1258 | 125 | 1338 | 1233, 1452 | 1.50 | 1.06, 2.10 | 0.020 |
| **KT**  Ratio × 1000 | 319 | 51.4 | 49.8, 53.1 | 115 | 54.3 | 51.4, 57.4 | 1.58 | 0.92, 2.69 | 0.095 |
| **MPO**  ng/mL | 224 | 5569 | 4867, 6372 | 84 | 7023 | 5632, 8757 | 1.18 | 0.97, 1.43 | 0.093 |
| **NEO**  nmol/L | 223 | 888 | 821, 960 | 82 | 861 | 744, 995 | 0.95 | 0.70, 1.30 | 0.749 |
| **REG1B**  ug/mL | 207 | 16.4 | 14.1, 19.1 | 76 | 16.9 | 13.1, 21.8 | 1.02 | 0.88, 1.19 | 0.810 |
| **sCD14**  pg/mL | 380 | 7.2x10^5 | 6.8 x10^5, 7.7x10^5 | 125 | 7.1x10^5 | 7.0 x10^5, 8.5  x10^5 | 1.17 | 0.86, 1.58 | 0.322 |

AAT = alpha-1 antitrypsin, CRP = C-reactive protein, CIT = citrulline, IFABP = intestinal fatty acid binding protein, KTR = kynurenine tryptophan ratio, MPO = myeloperoxidase, NEO = neopterin, REG1B = regenerating enzyme 1B, sCD14 = soluble CD14.

* Risk ratio corresponding to a unit increase (one natural log) in the biomarker.

**^&^** Geometric means are shown for biomarker values.

P values highlighted in **bold** remained significant after adjusting for multiple testing using the Benjamini-Hochberg procedure.

*Table S8-B*

| **Biomarker score** | **Seronegative** | | | **Seropositive** | | | **Unadjusted analysis** | | |
| --- | --- | --- | --- | --- | --- | --- | --- | --- | --- |
|  | **N** | **Mean** | **s.d.** | **N** | **Mean** | **s.d.** | **Unadj RR*** | **95% CI** | **P value** |
| **Permeability**  **(max=2)** | 220 | 0.99 | 0.70 | 83 | 1.00 | 0.72 | 1.01 | 0.76, 1.34 | 0.935 |
| **Damage**  **(max=6)** | 202 | 2.87 | 1.40 | 75 | 2.93 | 1.19 | 1.02 | 0.90, 1.17 | 0.727 |
| **Inflammation**  **(max=4)** | 223 | 1.99 | 1.00 | 82 | 2.02 | 0.99 | 1.03 | 0.86, 1.25 | 0.724 |
| **Translocation**  **(max=6)** | 319 | 2.96 | 1.39 | 115 | 3.17 | 1.36 | 1.08 | 0.97, 1.21 | 0.149 |
| **EED SHINE (max=18)** | 172 | 8.63 | 2.47 | 69 | 9.27 | 2.28 | 1.08 | 1.00, 1.17 | 0.058 |
| **EED Kosek (max=10)** | 219 | 4.94 | 2.40 | 81 | 5.12 | 2.41 | 1.03 | 0.95, 1.11 | 0.545 |

* Risk ratio corresponding to a unit increase in the EED biomarker score.

^#^ Models were adjusted for WASH arm, season of birth, breastfeeding status and weight-for-age Z-score around the time of vaccination.

P values highlighted in **bold** remained significant after adjusting for multiple testing using the Benjamini-Hochberg procedure.

**Table S9:** Baseline characteristics of infants, mothers and their households across WASH and non-WASH groups.

|  | **Non-WASH**  (Infants N=306) | **WASH**  (Infants N=199) | **All**  (Infants N=505) |
| --- | --- | --- | --- |
| **Infant characteristics**  Gender, % female  Birthweight, kilograms; mean (SD)  Low birthweight (<2.5kg), %  Institutional delivery, %  Normal Vaginal delivery, %  Born in rotavirus season, %  Exclusive breastfeeding, %  Receipt of concurrent OPV, % | 46.4  3.16 (0.4)  5.9  91.0  94.1  37.2  93.4  95.1 | 52.8  3.10 (0.5)  10.6  92.4  94.0  38.2  93.5  95.0 | 48.9  3.14 (0.5)  7.7  91.6  94.1  37.6  93.5  95.1 |
| **Maternal characteristics**  Age, years; mean (SD)  Parity, median (IQR)  Height, cm; mean (SD)  Mid upper arm circumference, cm; mean (SD)  Married, %  Completed years of schooling, median (IQR)  Unemployed, %  Religion:  Apostolic, %  Other Christian, %  Other religion, %  Wealth Quintile:  Lowest, %  Second ,%  Middle, %  Fourth, %  Highest, % | 26.8 (6.3)  2 (2, 2)  160.7 (5.8)  26.8 (2.9)  96.0  10 (9, 11)  91.4  46.2  36.1  17.7  17.1  19.9  23.6  21.6  17.8 | 27.4 (6.4)  2 (2, 2)  159.6 (5.8)  27.1 (3.8)  94.4  10 (9, 11)  86.7  47.4  33.0  19.6  15.4  21.5  24.1  16.9  22.1 | 27.0 (6.4)  2 (2, 2)  160.2 (5.8)  26.9 (3.3)  95.4  10 (9, 11)  89.6  46.7  34.9  18.4  16.4  20.5  23.8  19.7  19.5 |
| **Household characteristics**  Household size, median (IQR)  Electricity, %  Open defecation, %  Any latrine, %  Improved latrine, %  Improved water source, %  Handwashing station present, %  Improved floor, %  Owns chickens, %  Livestock observed in house, % | 5 (3, 6)  3.4  54.4  34.3  27.7  64.6  3.2  51.0  81.6  43.4 | 5 (4, 6)  1.0  37.2  51.9  49.2  71.2  23.4  56.5  81.0  48.5 | 5 (4, 6)  2.5  47.6  41.2  36.2  67.2  11.4  53.2  81.4  45.4 |

**Supplementary figures**

**Figure S1.** Frequency of infants according to a) number of biomarkers in top quartile and b) number of domains with at least one biomarker in the top quartile.

**Figure S2.** Scree plot and table showing principal components and their loading. Coefficients >0.4 or <-0.4 are shaded in grey.
